# Retraction: ‘MALAT1 promotes gastric adenocarcinoma through the MALAT1/miR-181a-5p/AKT3 axis’ (2019), by Lu *et al.*

**DOI:** 10.1098/rsob.230404

**Published:** 2023-11-03

**Authors:** Open Biology editorial team

**Keywords:** gastric adenocarcinoma, metastasis-associated lung adenocarcinoma transcript 1 (MALAT1), miR-181a-5p, RAC-γ serine/threonine-specific protein kinase (AKT3) pathway

*Open Biol.* **9**, 190095 (Published online 4 September 2019). (https://doi.org/10.1098/rsob.190095)

The Editor-in-Chief in agreement with the Publisher has retracted this article. Following an investigation, flaws and inconsistencies in the sequence analyses and western blots were found (figs. 4 and 5). The authors have not been able to supply the raw data as requested, thus, the editors consider the conclusions of this article to be invalid. The authors have not responded to correspondence about this retraction.

The image integrity standards and policies for the journal can be found here: https://royalsocietypublishing.org/doi/10.1098/rsob.200165.

Prof. Jon Pines FRS

Editor-in-Chief, *Open Biology.*

